# Author Correction: PEAR1 regulates expansion of activated fibroblasts and deposition of extracellular matrix in pulmonary fibrosis

**DOI:** 10.1038/s41467-022-35543-4

**Published:** 2022-12-14

**Authors:** Yan Geng, Lin Li, Jie Yan, Kevin Liu, Aizhen Yang, Lin Zhang, Yingzhi Shen, Han Gao, Xuefeng Wu, Imre Noth, Yong Huang, Junling Liu, Xuemei Fan

**Affiliations:** 1 Department of Biochemistry and Molecular Cell Biology, Shanghai Jiao Tong University School of Medicine, Shanghai, China; 2 College of Arts and Science, Vanderbilt University, Nashville, TN USA; 3 Cyrus Tang Hematology Center, Soochow University, Suzhou, China; 4 State Key Laboratory of Cognitive Neuroscience and Learning and Beijing Key Laboratory of Genetic Engineering Drugs & Biotechnology, College of Life Sciences, Beijing Normal University, Beijing, China; 5 Shanghai Institute of Immunology, Department of Immunology and Microbiology, Shanghai Jiao Tong University School of Medicine, Shanghai, China; 6 Department of Medicine, University of Virginia, Charlottestville, VA USA; 7 Shanghai Synvida Biotechnology Co., Ltd, Shanghai, China

**Keywords:** Target identification, Mechanisms of disease, Cystic fibrosis, Respiratory tract diseases

Correction to: *Nature Communications* 10.1038/s41467-022-34870-w, published online 19 November 2020

The original version of this article contained errors in Figure 3. In Figure 3e, the values on the heat range map were inverted, and read ‘2, 1, 0, -1, -2’, while they should read ‘-2, -1, 0, 1, 2’. In addition, the ontology in Fig. 3c contained multiple typographical errors, which read “Exracellular matrix orization” instead of “Extracellular matrix organization”, “Mesenchymedevelopment” instead of “Mesenchyme development”, “Owth factor stimulus” instead of “Response to growth factor stimulus”, “Collagen fibrillarorganization” instead of “Collagen fibril organization”.

These errors have been corrected in both the PDF and HTML versions of the article.

