# Supplementary material for: PEAR1 regulates expansion of activated fibroblasts and deposition of extracellular matrix in pulmonary fibrosis
Source: Nat Commun. 2022 Nov 19;13:7114. doi: 10.1038/s41467-022-34870-w (PMC9675736; doi:10.1038/s41467-022-34870-w)
Supplement: Supplementary file 1 — Supplementary Information [file 41467_2022_34870_MOESM1_ESM.pdf]

Supplementary figures and figure legends

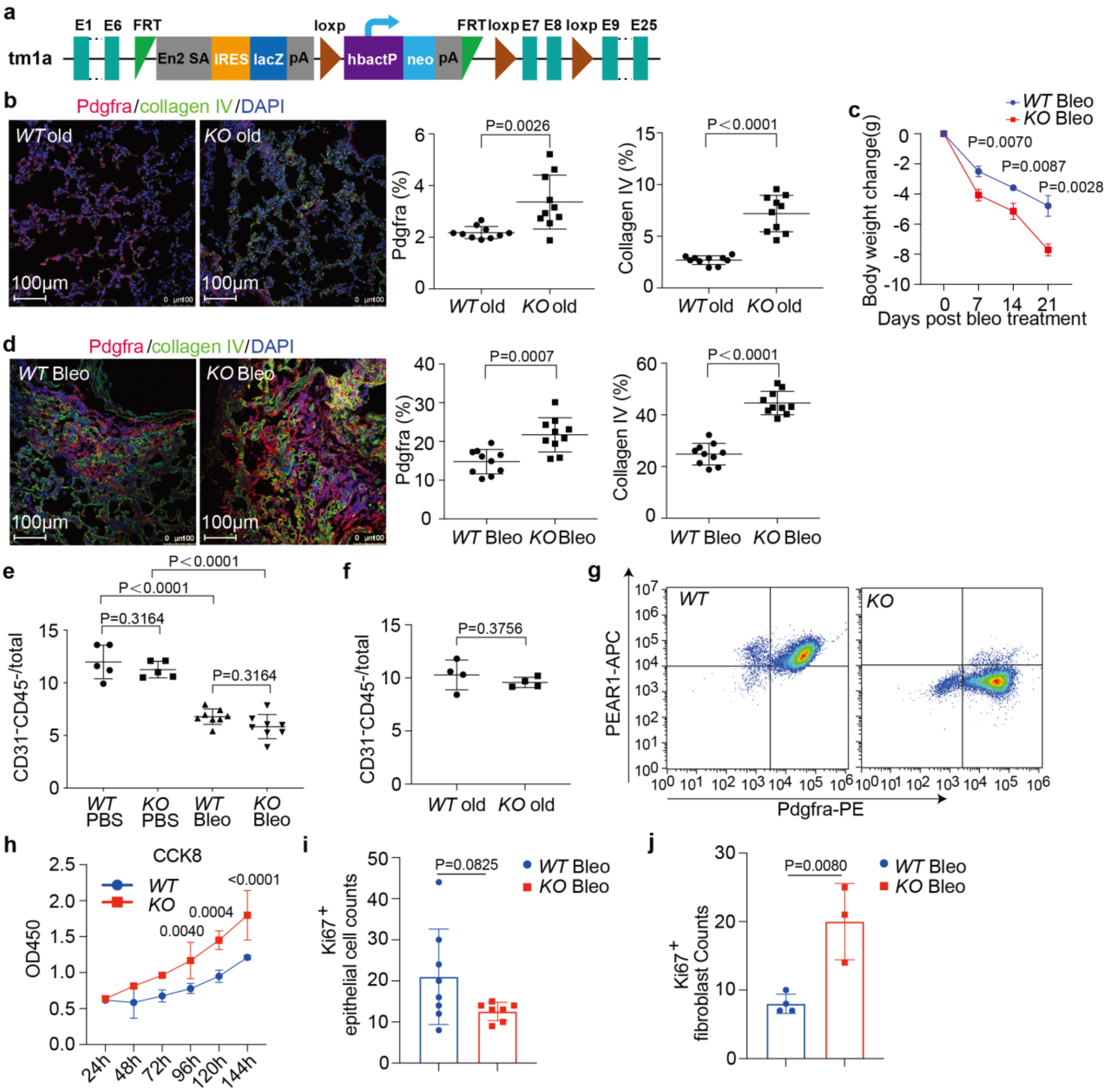

Supplementary Fig.1| *Pear1* deficiency exacerbated PF and promoted proliferation of mesenchymal cells. a, Schematic of the "knockout first" targeting strategy used for generating *Pear1*$^{-/-}$ mice. FRT: Flp-recombinase target, En2 SA: Engrailed-2 splice acceptor, pA: SV40 polyadenylation signal, neo: Neomycin resistance gene, IRES: internal ribosome entry site. **b,** Representative images of immunofluorescence staining on lung sections from 12-month-old *WT* mice and *Pear1*$^{-/-}$ mice for Pdgfra (red), collagen IV (green) and DAPI (blue). The fluorescence positive area was calculated (n=10 mice per group). (Scale bars, 100 μm). **c,** Body weight loss of *WT* and *Pear1*$^{-/-}$ mice induced by 2 μg/g bleo (n=10 mice per group). **d,** Representative images of immunofluorescence staining on lung sections from *WT* mice and *Pear1*$^{-/-}$ mice on day 21 after bleo treatment for Pdgfra (red), collagen IV (green) and DAPI (blue). The fluorescence positive area was calculated (n=10 mice per group). (Scale bars, 100 μm). **e,** The proportion of CD45^{-}CD31^{-} cells were analyzed in the lung tissue of *WT* and *Pear1*$^{-/-}$ mice with or without treatment of bleo (n=5 mice in PBS group; n=8 mice in bleo group). **f,** The proportion of CD45^{-}CD31^{-} cells were analyzed in the lung tissue of 12 month old *WT* and *Pear1*$^{-/-}$ mice (n=4 mice in each group). **g,** The expression of PEAR1 in cultured primary fibroblasts isolated from *WT* and *Pear1*$^{-/-}$ lung tissue was detected by flow cytometery with the APC anti-mouse PEAR1 antibody and PE anti-Pdgfra antibody. **h,** CCK8 assay of culture fibroblasts isolated from *WT* and *Pear1*$^{-/-}$ mice. **i, j,** Ki67 staining was performed in lung sections from *WT* and *Pear1*$^{-/-}$ mice (n=8 mice in *WT* and n=8 mice in *Pear1*$^{-/-}$ group of Ki67^{+} epithelial cell counts; n=4 mice in *WT* and n=3 mice in *Pear1*$^{-/-}$ group

of Ki67^{+} fibroblast counts). The podoplanin, Pdgfra was co-stained for indicating epithelial cells (**i**) and fibroblasts (**j**), respectively. For **b**, **c**, **f**, **h**, **i**, **j**, two-tailed t test was used. For **e**, one-way ANOVA was used. Data are presented as mean ± SD. Source data are provided as a Source Data file.

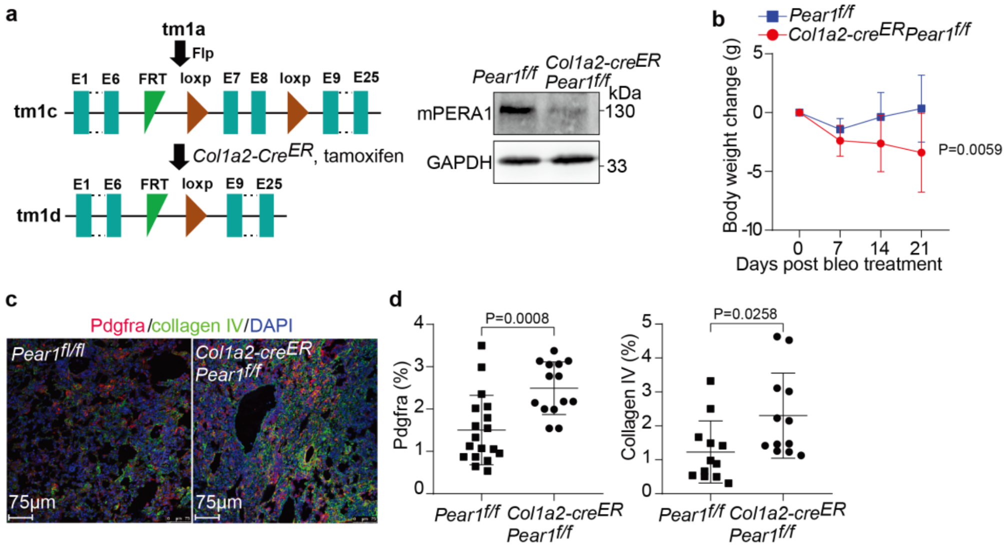

Supplementary Fig.2| *Pear1* deficiency exacerbate PF by direct regulation of mesenchymal cells function. a, Construction of mesenchymal cell specific *Pear1* knockout mice. *Pear1*$^{tm1c/+}$ (*Pear1*$^{fl/wt}$) allele was produced by crossing *Pear1*$^{tm1a/tm1a}$ with a globe Flp transgenic mouse strain. *Pear1*$^{tm1d/+}$ (*Col1a2-cre*^{ER}*Pear1*$^{fl/wt}$) allele was generated by mating *Pear1*$^{tm1c/tm1c}$ with a *Col1a2-cre*^{ER} transgenic mouse strain. After 7 days post tamoxifen-inducing, the deficiency of PEAR1 in lung fibroblasts was evaluated by western blotting. The experiments were repeated three times and the results were similar. **b,** Body weight loss of *Pear1*$^{f/f}$ and *Col1a2-Cre*^{ER}*Pear1*$^{f/f}$ mice induced by 1.5 μg/g bleo through endotracheal atomization (n=7 mice in *Pear1*$^{f/f}$ group; n=5 mice in *Col1a2-Cre*^{ER}*Pear1*$^{f/f}$ group). **c,** Representative images of immunofluorescence staining on lung sections from *Pear1*$^{f/f}$ and *Col1a2-Cre*^{ER}*Pear1*$^{f/f}$ mice on day 21 after bleo treatment for Pdgfra (red), collagen IV (green) and DAPI (blue). (Scale bars, 75 μm). **d,** The fluorescence positive area of Pdgfra and collagen IV was calculated by Image J software and statistics were performed using GraphPad (n=10 mice per group). For **b**, **d**, two-tailed t test was used. Data are presented as mean ± SD. Source data are provided as a Source Data file.

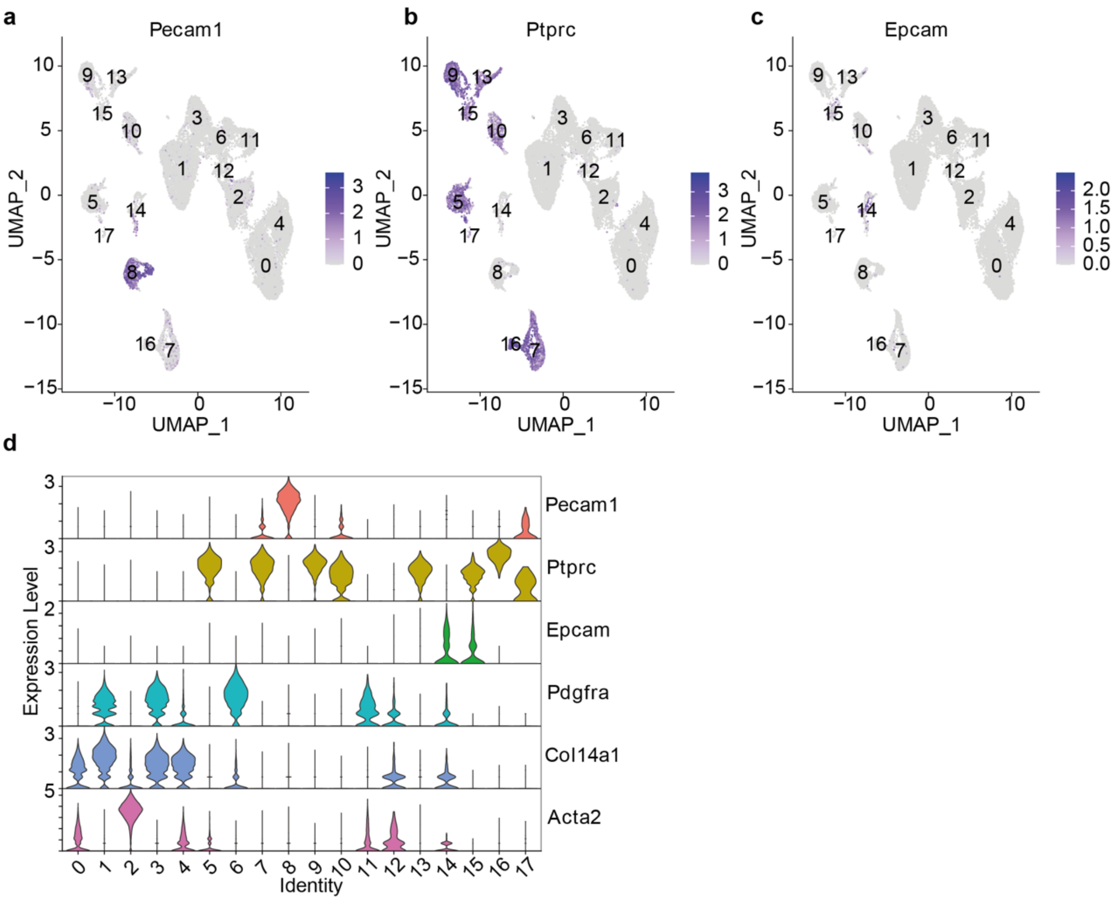

Supplementary Fig. 3| Evaluation of mesenchymal cell selections. **a-d**, Flow cytometry with three markers, Pecam1 (CD31), Ptprc (CD45) and Epcam (CD326) was used to filter out positive cells. Cells without the markers were used for scRNA-seq. Leakage for cell filtering was found in Cluster 8 for CD31 (**a**), Cluster 5, 7, 9, 10, 13, 15, 16, 17 for CD45 (**b**), and Cluster 14 and 15 for CD326 (**c**). The rest clusters were confirmed to be negative with the three markers, but positive with markers for mesenchymal cells (**d**).

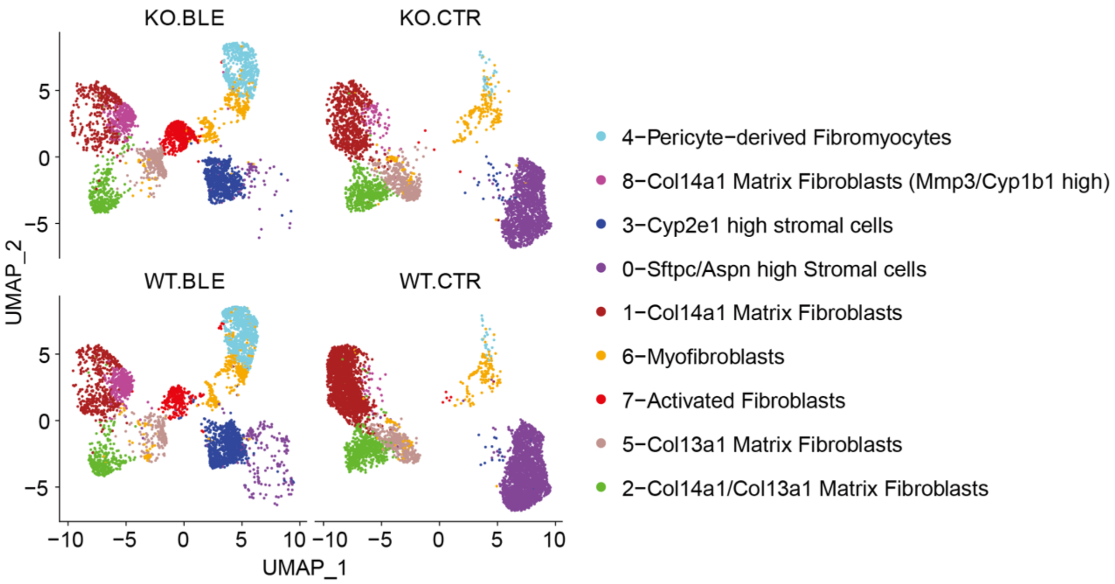

Supplementary Fig. 4| UMAP of cell clusters for each experiment condition. Significantly enlarged cell clusters 3, 4, 7 were identified in bleo-treated animals compared to controls.

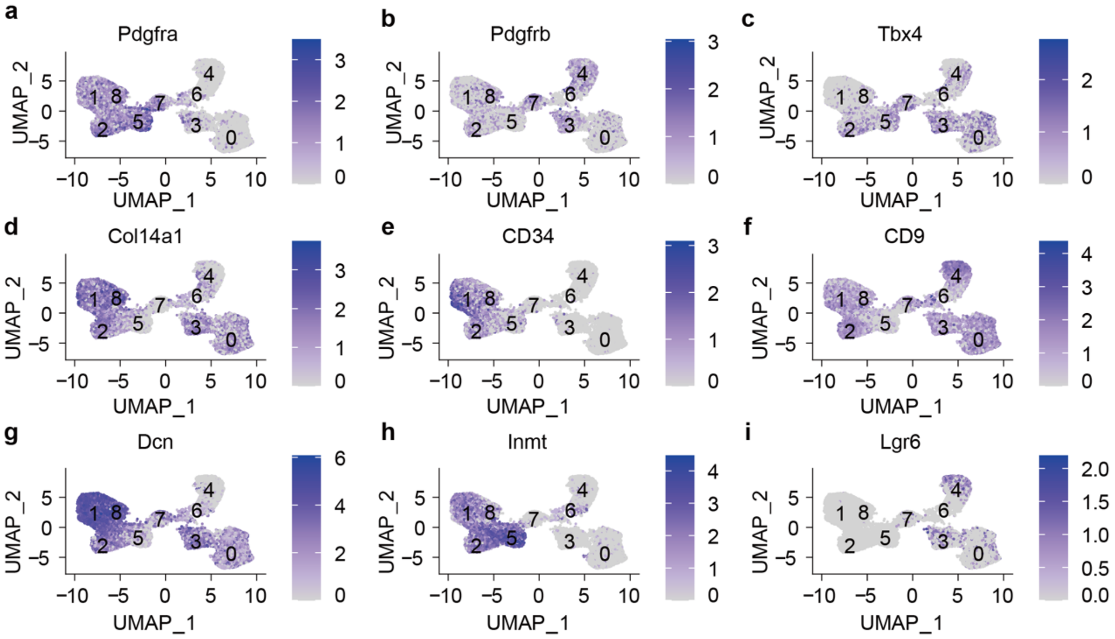

Supplementary Fig. 5| UMAP of Feature plot of universal mesenchymal cell (MSC) markers. a-i, MSC markers in each cluster of the combined samples. Blue bar on left side of each plot represents the scaled expression levels of the corresponding gene. Annotation of cell clusters: 0-Sftpc/Aspn high Stromal cells, 1-Col14a1 Matrix Fibroblasts, 2-Col14a1/Col13a1 Matrix Fibroblasts, 3-Cyp2e1 high stromal cells, 4-Pericyte-derived Fibromyocytes, 5-Col13a1 Matrix Fibroblasts, 6-Myofibroblasts, 7-Activated Fibroblasts, 8-Col14a1 Matrix Fibroblasts (Mmp3/Cyp1b1 high).

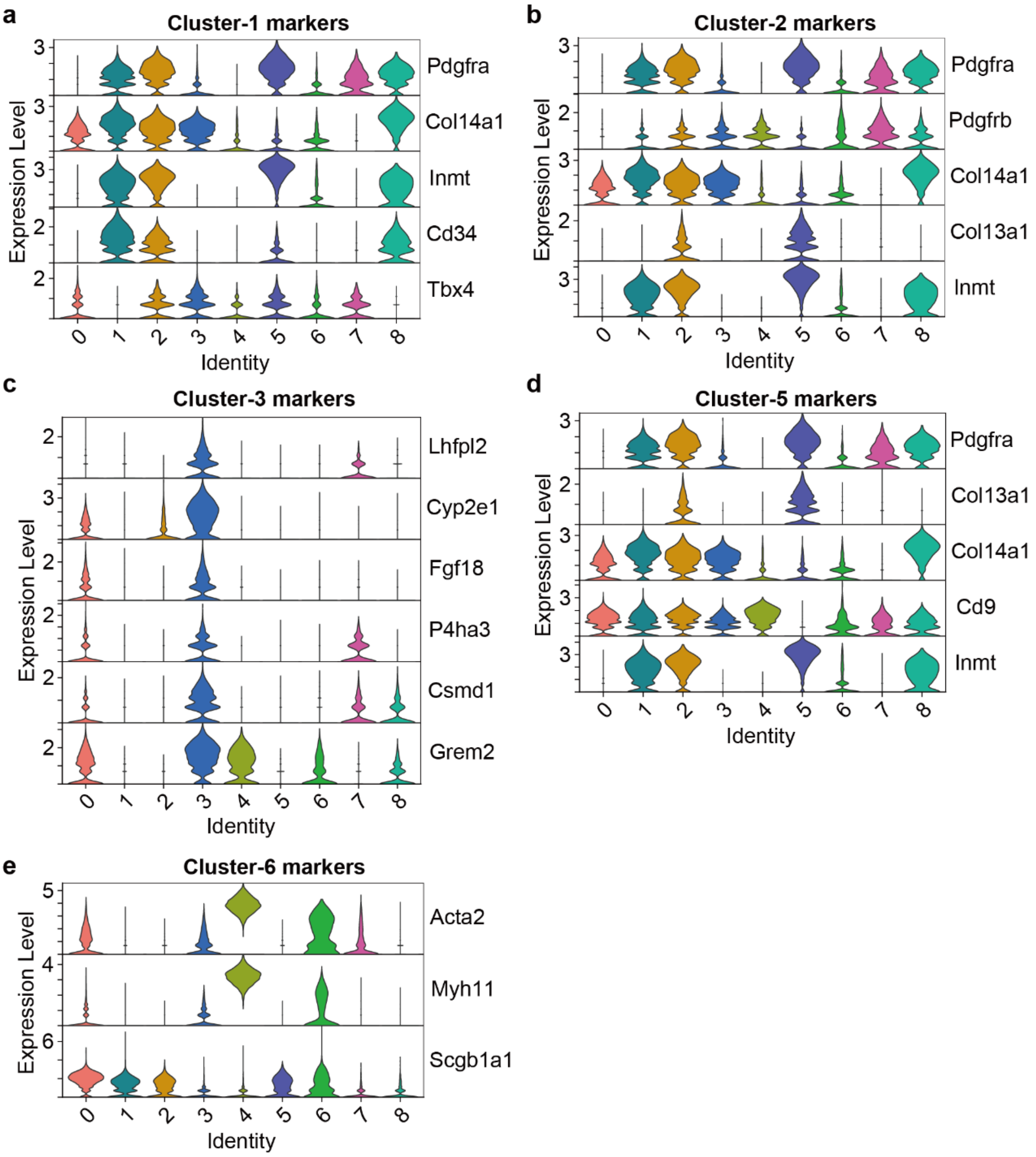

Supplementary Fig. 6| Violin plot of the expression levels of cluster markers. a-e, Markers for Cluster 1, 2, 3, 5, 6. Y-axis indicates the scaled expression levels of the corresponding genes. X-axis represents cell clusters: 0-Sftpc/Aspn high Stromal cells, 1-Col14a1 Matrix Fibroblasts, 2-Col14a1/Col13a1 Matrix Fibroblasts, 3-Cyp2e1 high stromal cells, 4-Pericyte-derived Fibromyocytes, 5-Col13a1 Matrix Fibroblasts, 6-Myofibroblasts, 7-Activated Fibroblasts, 8-Col14a1 Matrix Fibroblasts (Mmp3/Cyp1b1 high).

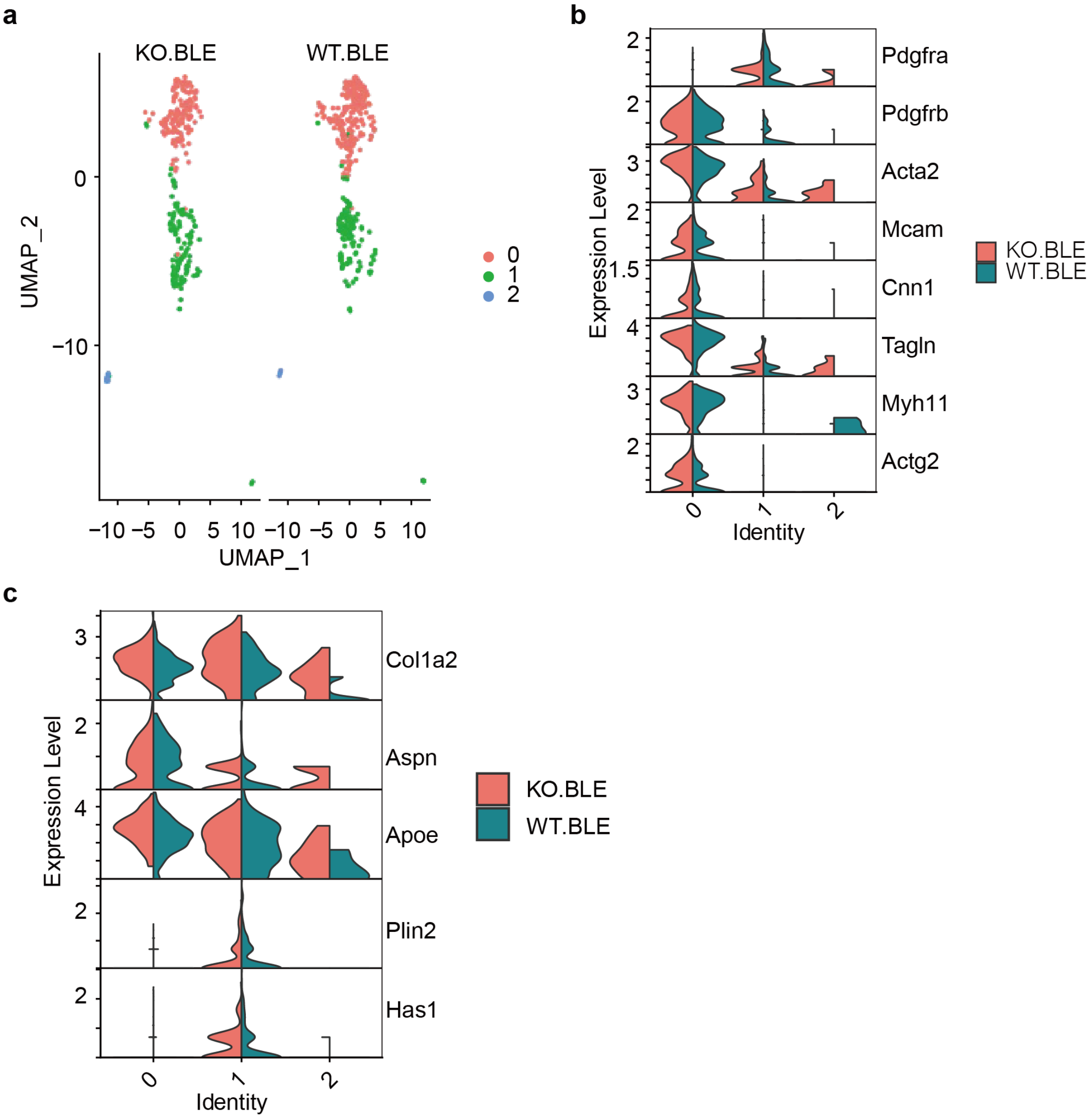

Supplementary Fig. 7| Subcluster and annotation of cluster 6. a, Two major subclusters (0 & 1) were identified in cluster 6. The percentage of each subclusters in the corresponding samples are shown on the right side. **b**, markers for subcluster 0; **c**, markers for subcluster 1.

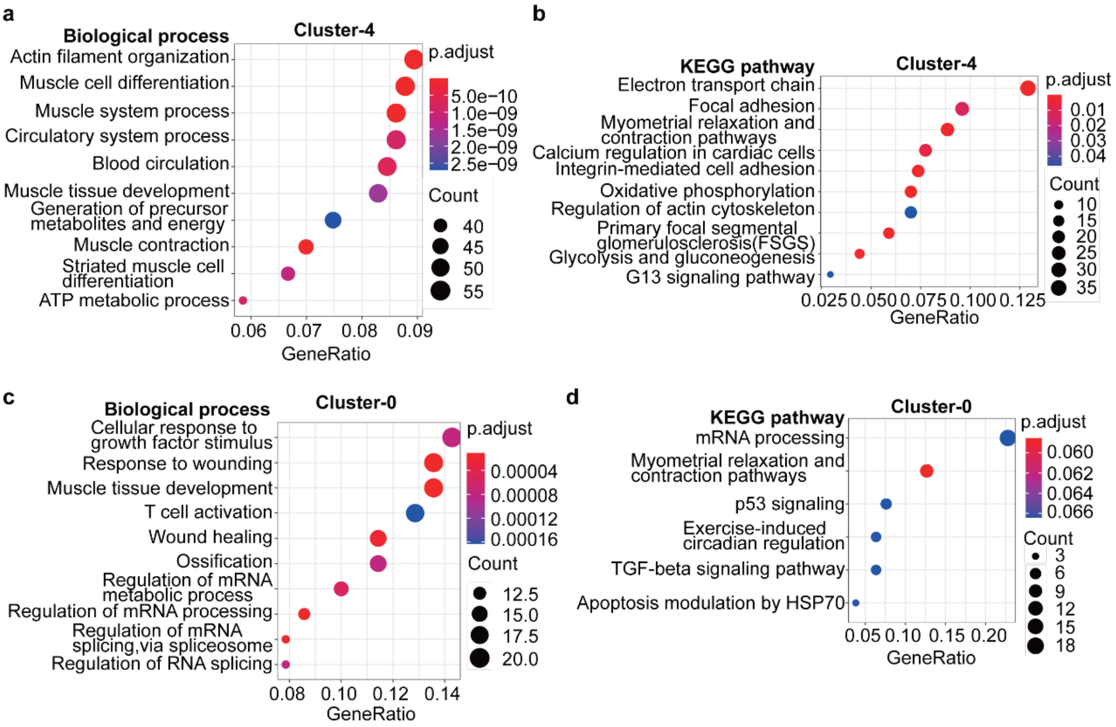

Supplementary Fig. 8| Functional enrichment analysis of cluster 4 genes and cluster 0 genes. a, b, Top 10 significant GO biological process (**a**) and top 10 significant KEGG pathways (**b**) of cluster 4 genes in *Pear1*$^{-/-}$ Bleo mice. **c, d,** Top 10 significant GO biological process (**c**) and top 10 significant KEGG pathways (**d**) of cluster 0 genes in *WT* Ctrl mice. Pathway overrepresentation analysis was performed using one-sided Fisher's exact test. Multiple comparisons were adjusted using Benjamini-Hochberg method, as indicated by the p.adjust on right panel.

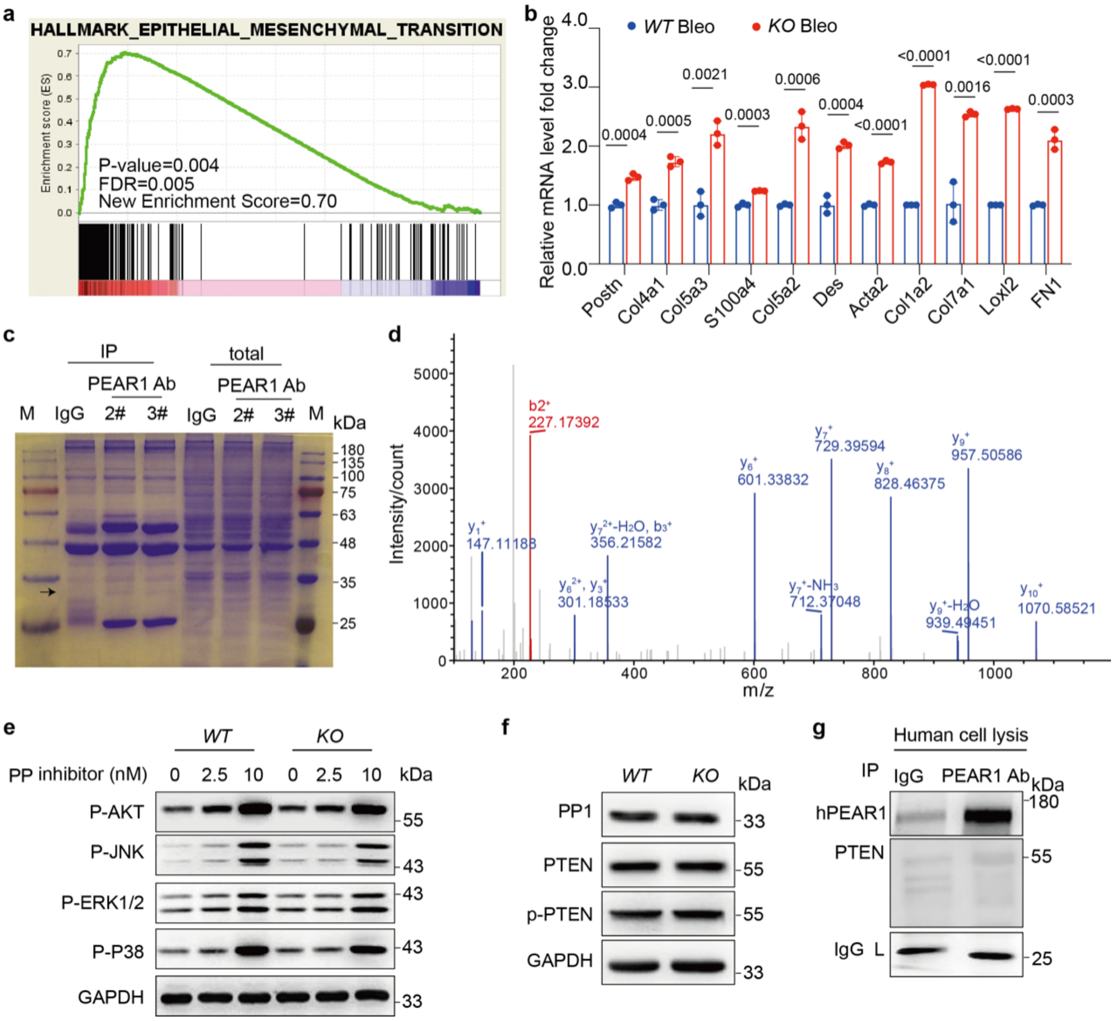

Supplementary Fig. 9| PEAR1 associated with PP1 to suppress fibrotic factor induced fibroblast activation. **a,** EMT markers were significantly enriched in $Pear1^{-/-}$ (KO) Bleo compared to *WT* Bleo (FDR=0.005). **b,** Confirmation of the increased expression levels of EMT markers by qPCR in $Pear1^{-/-}$ Bleo compared to *WT* Bleo. **c,** The possible associated proteins of PEAR1 in fibroblast was studied by immunoprecipitation of PF fibroblasts with anti-PEAR1 antibody. The different bands below 63kd and around 35kD between IgG control and Pear1 antibody in Coomassie brilliant blue staining were cut off and the protein were identified by mass spectrometry. The experiments were repeated three times and the results were similar. **d,** Mass spectrometric identification of protein phosphatase 1 catalytic subunit alpha (PP1α). **e,** The phosphorylation levels of AKT, P38 ERK1/2 and JNK1/2 were evaluated in cultured fibroblasts isolated from *WT* and $Pear1^{-/-}$ mice incubated with 2.5 nM or 10 nM PP1 inhibitor for 15 minutes, respectively. The experiments were repeated three times and the results were similar. **f,** The levels of PP1, PTEN, p-PTEN were evaluated in cultured fibroblasts isolated from *WT* and $Pear1^{-/-}$ mice. The experiments were repeated three times and the results were similar. **g,** No binding between PEAR1 and PTEN were detected by immunoprecipitation in cultured PF fibroblasts. The experiments were repeated three times and the results were similar. For **a**, one-sided Fisher's exact test was used. For **b**, two-tailed t test was used. Data are presented as mean ± SD. Source data are provided as a Source Data file.

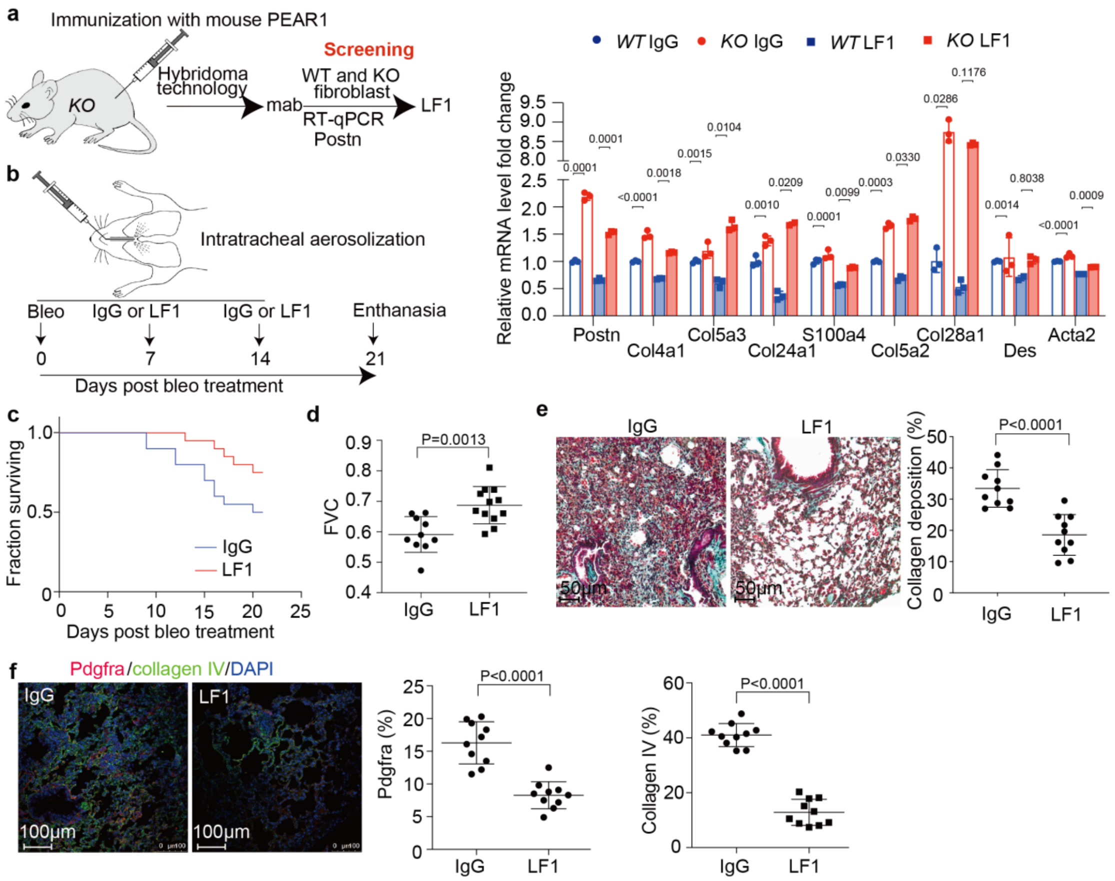

Supplementary Fig. 10| Monoclonal antibody target mouse PEAR1 for PF therapy. a, Preparation and screening strategies of anti-mouse PEAR1 monoclonal antibodies. An effective monoclonal antibody named as LF1 was identified, and qPCR results showed that LF1 can effectively inhibit the synthesis of extracellular matrix proteins in pulmonary fibroblasts in a PEAR1-dependent manner (n=3 biologically independent samples in each group). **b,** A flow chart of evaluating the roles of LF1 in bleo-induced lung fibrosis in *vivo*. LF1 is mouse IgG1 isotype, and mouse IgG1 was used as a negative control. **c,** The survival curves of *WT* mice treated with 1 mg/kg anti-PEAR1 monoclonal antibody LF1 induced by 1.5 μg/g bleo through endotracheal atomization (n=20 mice per group). **d,** FVC was measured in *WT* mice treated with LF1 or IgG on day 21 after bleo treatment (n=10 mice in IgG group; n=12 mice in anti-PEAR1 group). **e,** Representative images of masson staining on lung sections from *WT* mice treated with LF1 on day 21 after bleo treatment. Green represents collagen deposition. The collagen area was calculated by Image J software and statistics were performed using GraphPad (n=10 mice per group). (Scale bars, 50 μm). **f,** Representative images of immunofluorescence staining on lung sections from *WT* mice treated with LF1 on day 21 after bleo treatment for Pdgfra (red), collagen IV (green) and DAPI (blue). (Scale bars, 100 μm). The fluorescence positive area was calculated by Image J software and statistics were performed using GraphPad (n=10 mice per group). For **d-f**, two-tailed t test was used. Data are presented as mean ± SD. Source data are provided as a Source Data file.

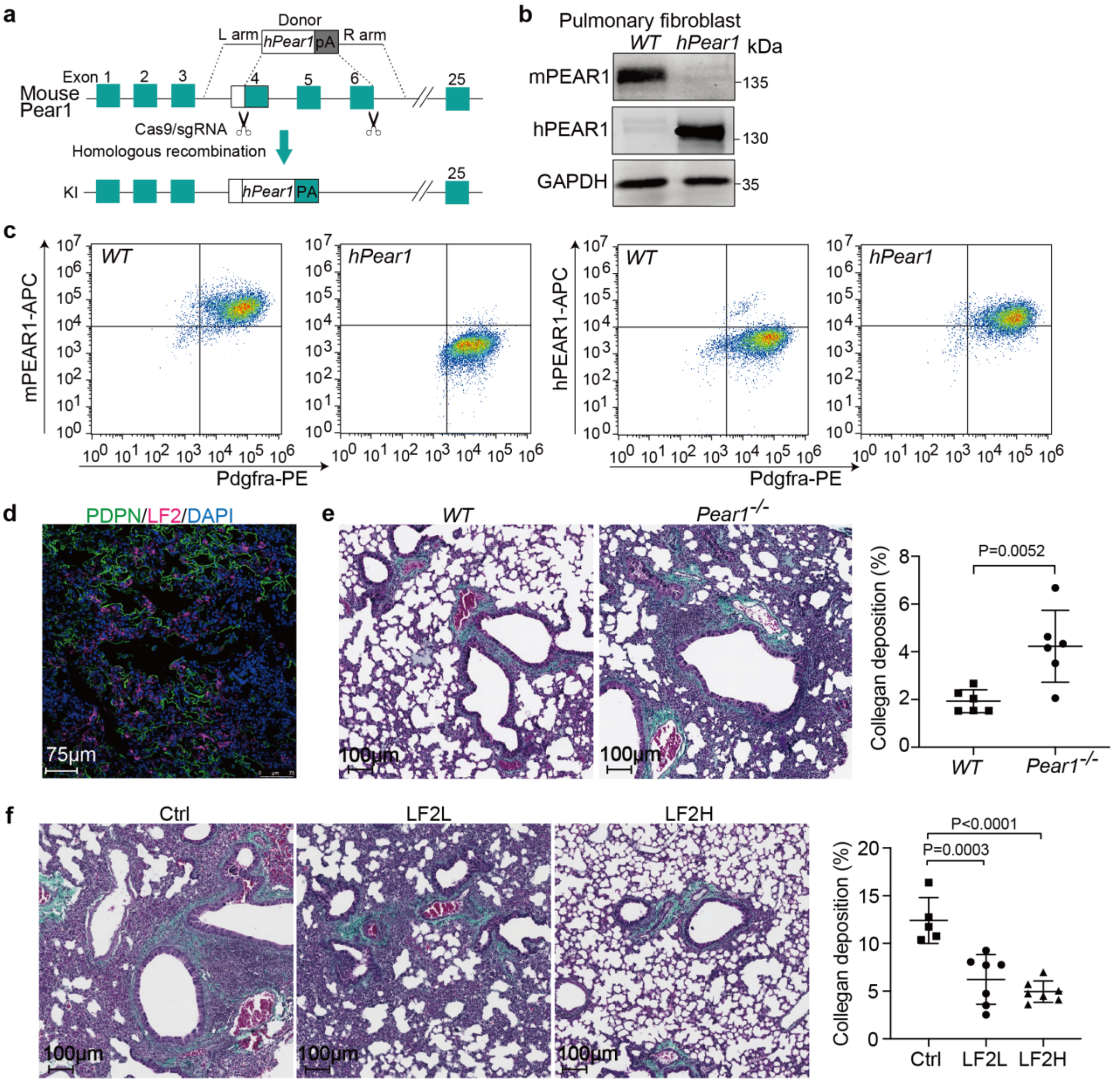

Supplementary Fig. 11| Construction of human *Pear1* transgenic mouse and evaluation of the function of LF2 in PF. a, Schematic diagram of humanized Pear1 mouse construction. The exon 4-6 of mouse *Pear1* was replaced by human *Pear1* cDNA, and SV40 poly A transcription termination signal was added after human *Pear1* cDNA to ensure that mouse *Pear1* is completely replaced by human *Pear1*. **b, c,** The expression levels of human PEAR1 and mouse PEAR1 in lung fibroblasts from humanized *Pear1* mice were detected by western blot **(b)** and flow cytometry **(c)**. The experiments were repeated three times and the results were similar. **d,** Representative image of the distribution of LF2. Intratracheal aerosolization of VivoTag 645-labeled LF2 (1mg/kg) into the mice on day 14 after bleo treatment. The lungs were obtained at 48h after LF2 administration, and the sections were co-stained with PDPN for indicating epithelial cells and DAPI. (Scale bars, 75 μm). **e,** Representative images of masson staining on lung sections from *WT* mice and *Pear1*$^{-/-}$ mice on day 23 after 0.4 mg/kg amiodarone administration. Green represents collagen deposition. The collagen area was calculated by Image J software and statistics were performed using GraphPad (n=6 mice per group). (Scale bars, 100 μm). **f,** Representative images of masson staining on lung sections from Ctrl (Saline) group mice, LF2L (0.5 mg/kg) and LF2H (1 mg/kg) group mice on day 23 after 0.8 mg/kg amiodarone administration. The collagen area (green) was calculated by Image J software and statistics were performed using GraphPad (n=5 mice in Ctrl group; n=7 mice in LF2L group; n=7 mice in LF2H group). (Scale bars, 100 μm). For **e**, two-tailed t test was used. For **f**, one-way ANOVA was used. Data are presented as mean ± SD. Source data are provided as a Source Data file.

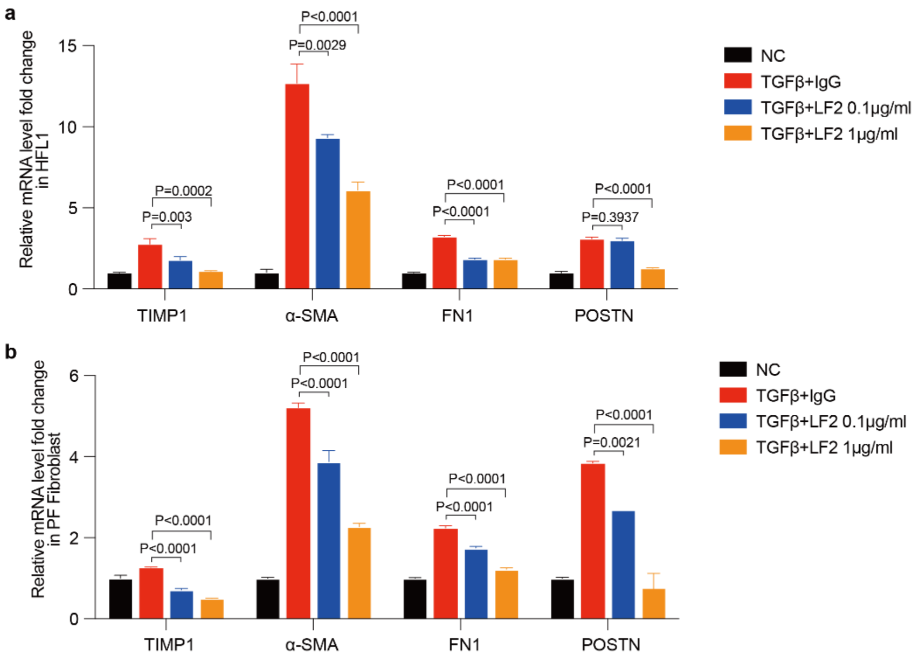

Supplementary Fig. 12| The function of LF2 on HFL1 and PF fibroblasts. a, b, Relative mRNA expression levels of ECM genes in HFL1 (**a**) and PF fibroblasts (**b**) incubated with 0.1 μg/mL or 1μg/mL LF2 and stimulated by TGFβ for 48 hours (n=3 biologically independent samples in each group). Human IgG4 was used as an isotype control for LF2. For **a**-**b**, one-way ANOVA was used. Data are presented as mean ± SD. Source data are provided as a Source Data file.

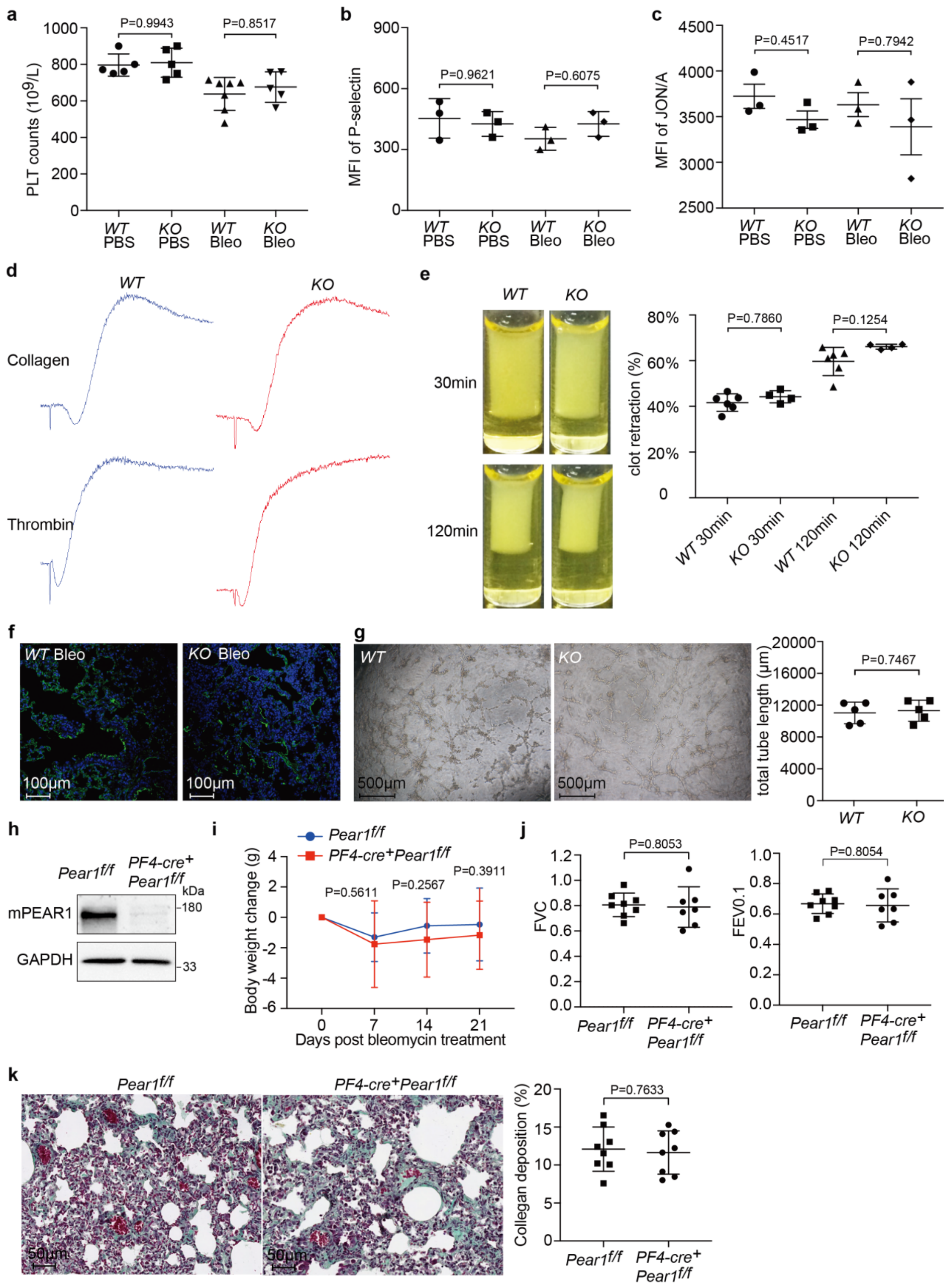

Supplementary Fig. 13| *Pear1* deficiency did not affect platelet activity and tubulogenesis of pulmonary endothelial cells. a, The platelet counts of peripheral blood obtained from normal or 2 μg/g bleo-induced *WT* and $Pear1^{-/-}$ mice (n=5 mice in *WT* PBS, $Pear1^{-/-}$ PBS, $Pear1^{-/-}$ Bleo group; n=7 mice in *WT* Bleo group). **b,c,** Platelet activity in normal or bleo-induced *WT* and $Pear1^{-/-}$ mice was measured by P-selectin exposure (b) and JON/A binding (c) with flow cytometry (n=3 mice in each group). **d,** Platelet activity in *WT* and $Pear1^{-/-}$ mice was measured by platelet aggregation in response to 1.5 μg/mL collagen and 0.05 U/mL thrombin. **e,** Platelet function in *WT* and $Pear1^{-/-}$ (KO) mice was evaluated by clot retraction. **f,** The morphology of pulmonary vessels in *WT* and $Pear1^{-/-}$

mice with PF were analyzed by immunofluorescence staining of CD31. (Scale bars, 100 μm). The experiments were repeated three times and the results were similar. **g,** Tube formation of the endothelial cell from *WT* and *Pear1*$^{-/-}$ mice in Matrigel matrix were detected. The whole plate was photographed and the angiogenesis analyser of NIH Image J software was used for statistical analysis of the tube length (n=5 images in each group). (Scale bars, 500 μm). **h,** The expression of PEAR1 in platelets isolated from *Pear1*$^{f/f}$ and *PF4-Cre*$^{+}$*Pear1*$^{f/f}$ mice was measured by western blotting. The experiments were repeated three times and the results were similar. **i,** Body weight loss of *Pear1*$^{f/f}$ and *PF4-Cre*$^{+}$*Pear1*$^{f/f}$ mice induced by 1.5 μg/g bleo (n=8 mice in *Pear1*$^{f/f}$ group; n=7 mice in *PF4-Cre*$^{+}$*Pear1*$^{f/f}$ group). **j,** FVC and FEV0.1 were measured of *Pear1*$^{f/f}$ and *PF4-Cre*$^{+}$*Pear1*$^{f/f}$ mice on day 21 after bleo treatment (n=8 mice in *Pear1*$^{f/f}$ group; n=7 mice in *PF4-Cre*$^{+}$*Pear1*$^{f/f}$ group) **k,** Representative images of masson staining on lung sections from *Pear1*$^{f/f}$ and *PF4-Cre*$^{+}$*Pear1*$^{f/f}$ mice on day 21 after bleo treatment. The collagen area (green) was calculated (n=8 mice in each group). (Scale bars, 50 μm). For **g**, **i**, **j**, **k**, two-tailed t test was used. For **a-c**, **e**, one-way ANOVA was used. Data are presented as mean ± SD. Source data are provided as a Source Data file.

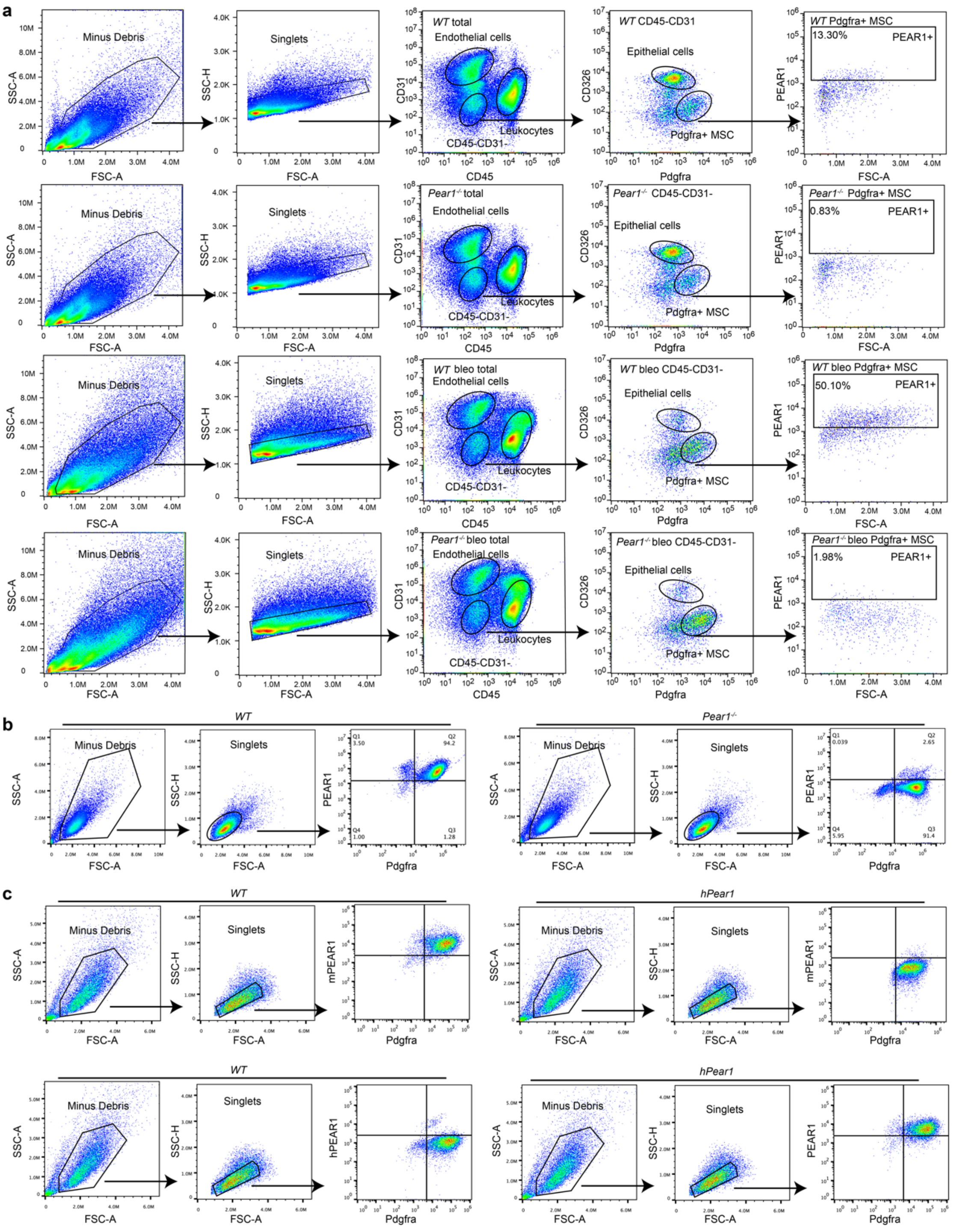

Supplementary Fig. 14| The gating strategy of flow cytometry. a, FACS plots showing the gating strategy to isolate endothelial cells, leukocytes, epithelial cells, pdgfr^{+} MSCs and PEAR1^{+} pdgfr^{+} MSCs. **b,c,** FACS plots showing the gating strategy to isolate single cell of cultured fibroblasts.

Supplementary Table 1. The primer information.

	Name	Forward (5'-3')	Reverse (5'-3')
QPCR Primer	Mouse Postn	GGGGTTGTCACTGTGAACTG	CGGCTGCTCTAAATGATGAA
	Mouse Col4a1	CTGGCACAAAAGGGACGAG	ACGTGGCCGAGAATTTCACC
	Mouse Col5a3	GGCAAAGATGGTATTCCAGGACC	TGCTTCCTTTGTGACCAGGCATC
	Mouse Col24a1	CCAGGTCGAAAAGGGTATATGGG	CGGTTATCCCTACTTCTCCAGG
	Mouse S100a4	AGCTCAAGGAGCTACTGACCAG	GCTGTCCAAGTTGCTCATCACC
	Mouse Col5a2	GTGGCATAGGAGAGAAAGGTGC	GCCAACTAAGCCTCTAGGACCA
	Mouse Col28a1	GCCTGGTCTTAAAGGAGAACCTG	AGCCATCACCTTTGAGTCCTGG
	Mouse Des	GTGGATGCAGCCACTCTAGC	TTAGCCGCGATGGTCTCATAC
	Mouse Acta2	TGCTGACAGAGGCACCACTGAA	CAGTTGTACGTCCAGAGGCATAG
	Mouse Loxl2	ATTAACCCCAACTATGAAGTGCC	CTGTCTCCTCACTGAAGGCTC
	Mouse Fn1	ATGTGGACCCCTCCTGATAGT	GCCCAGTGATTTCAGCAAAGG
	Mouse 18sRNA	TTGACTCAACACGGGAAACC	AGACAAATCGCTCCACCAAC
	Human POSTN	CAGCAAACCACCTTCACGGATC	TTAAGGAGGCGCTGAACCATGC
	Human COL8A1	AGGAAGCCGTACCCAAGAAAGG	GGTATCCCATGACCTGGCAAAC
	Human COL1A1	TAGGGTCTAGACATGTTCAGCTTTGT	GTGATTGGTGGGATGTCTTCGT
	Human COL5A3	GTGGCCGTCAGCATAGATGG	TGAATGTCTCCCTCGAAAGTCTT
	Human DES	TCCAGTCCTACACCTGCGAGAT	CGCAATGTTGTCCTGGTAGCCA
	Human COL4A1	TGTTGACGGCTTACCTGGAGAC	GGTAGACCAACTCCAGGCTCTC
	Human COL3A1	CCCACTATTATTTTGGCACAACAG	AACGGATCCTGAGTCACAGACA
	Human COL5A2	TAGGAACTGATGGTACTCCTGG	GGCCTATCGGACCCTGAATAC
	Human COL7A1	TTACGCCGCTGACATTGTGTT	ACCAGCCCTTCGAGAAAGC
	Human FN1	CGGTGGCTGTCAGTCAAAG	AAACCTCGGCTTCCTCCATAA
	Human TIMP1	GGAGAGTGTCTGCGGATACTTC	GCAGGTAGTGATGTGCAAGAGTC
	Human ACTA2	CTATGAGGGCTATGCCTTGCC	GCTCAGCAGTAGTAACGAAGGA
	Human 18SRNA	GAGCGGTCGGCGTCCCCCAACTTC	GCGCGTGCAGCCCCGGACATCTAA
Genotype Primer	*Pear1*$^{tm1a/+}$-KO	GCTACTGTCTGCTCCTTCAGTTCCC	CTCCTACATAGTTGGCAGTGTTTGG
	Pear1$^{tm1a/+}$-WT	GCTACTGTCTGCTCCTTCAGTTCCC	GCAACTCAGGGTTAGACTGGGTAGG
	Pear1$^{tm1c/+}$	GTCTGCTCCTTCAGTTCCCC	GAGGGAAGGGTTGTGTCAGG
	Flp	CACTGATATTGTAAGTAGTTTGC	CTAGTGCGAAGTAGTGATCAGG
	Col1a2-cre^{ER}-WT	ATTATTTTAGCACCACGGCAGC	TTCTCCGACAGATTAGAGGGCGAC
	Col1a2-cre^{ER}	ATCGTCCTTTCCTTGGCTGCTC	TTCTCCGACAGATTAGAGGGCGAC
	PF4-cre	CCCATACAGCACACCTTTTG	TGCACAGTCAGCAGGTT
	hPear1-LD	GCAGTGGGCTGATTTCTTC	TGCTACAGTTGACTCCCCAT
	hPear1-RD	GGCAGTTTGGAGAAGGTTG	CCAACCCCAGCTACAGTTA
	hPear1-WT	GCAGTGGGCTGATTTCTTC	CCAACCCCAGCTACAGTTA
