# Supplementary material for: PEAR1 regulates expansion of activated fibroblasts and deposition of extracellular matrix in pulmonary fibrosis
Source: Nat Commun. 2022 Nov 19;13:7114. doi: 10.1038/s41467-022-34870-w (PMC9675736; doi:10.1038/s41467-022-34870-w)
Supplement: Supplementary file 3 — Description of Additional Supplementary Files [file 41467_2022_34870_MOESM3_ESM.pdf]

Description of Additional Supplementary Files:

Supplementary Data 1: DEGs list. Pdgfra+ cells were selected for bulk RNA-seq assay. Differentially expressed genes (DEGs) between two experiment conditions were identified using criteria of fold change>2 and FDR<0.05. For two-group comparisons, the empirical Bayes (eBayes) moderated two-sided t-test was used and adjusted using Benjamini-Hochberg method, as shown in the column M (padj). T-statistic are shown in column K (stat).
