# Supplementary material for: PEAR1 regulates expansion of activated fibroblasts and deposition of extracellular matrix in pulmonary fibrosis
Source: Nat Commun. 2022 Nov 19;13:7114. doi: 10.1038/s41467-022-34870-w (PMC9675736; doi:10.1038/s41467-022-34870-w)
Supplement: Supplementary file 5 — Reporting Summary [file 41467_2022_34870_MOESM5_ESM.pdf]

nature portfolio

Corresponding author(s): Xuemei Fan, Junling Liu

Last updated by author(s): Nov 2, 2022

Reporting Summary

Nature Portfolio wishes to improve the reproducibility of the work that we publish. This form provides structure for consistency and transparency in reporting. For further information on Nature Portfolio policies, see our Editorial Policies and the Editorial Policy Checklist.

Statistics

For all statistical analyses, confirm that the following items are present in the figure legend, table legend, main text, or Methods section.

n/a	Confirmed	
☐	☒	The exact sample size (*n*) for each experimental group/condition, given as a discrete number and unit of measurement
☐	☒	A statement on whether measurements were taken from distinct samples or whether the same sample was measured repeatedly
☐	☒	The statistical test(s) used AND whether they are one- or two-sided<br>*Only common tests should be described solely by name; describe more complex techniques in the Methods section.*
☒	☐	A description of all covariates tested
☒	☐	A description of any assumptions or corrections, such as tests of normality and adjustment for multiple comparisons
☐	☒	A full description of the statistical parameters including central tendency (e.g. means) or other basic estimates (e.g. regression coefficient) AND variation (e.g. standard deviation) or associated estimates of uncertainty (e.g. confidence intervals)
☐	☒	For null hypothesis testing, the test statistic (e.g. *F*, *t*, *r*) with confidence intervals, effect sizes, degrees of freedom and *P* value noted<br>*Give P values as exact values whenever suitable.*
☒	☐	For Bayesian analysis, information on the choice of priors and Markov chain Monte Carlo settings
☒	☐	For hierarchical and complex designs, identification of the appropriate level for tests and full reporting of outcomes
☒	☐	Estimates of effect sizes (e.g. Cohen's *d*, Pearson's *r*), indicating how they were calculated

Our web collection on statistics for biologists contains articles on many of the points above.

Software and code

Policy information about availability of computer code

Data collection	1.Flow cytometry data was collected on CytoFLEX (Beckman Coulter).<br>2.sc-RNA seq data was collected by a Chromium Controller instrument (10x Genomics) and NovaSeq platform (Illumina).<br>3.Bulk tissue data was collected by HiSeq X10 from Illumina.<br>4.Pulmonary function data was collected by anires2005 pulmonary function analysis system (version 2.0, bestlab, China).<br>5. Platelet aggregation data was collected by an aggregometer (Chrono-Log) according to the manufacturer's protocol.

Data analysis	Flow cytometry data was analyzed using FlowJo (v10.0.7) software. The confocal images, clot retraction and tube length were analyzed using Image J (v1.51). We used Cell Ranger version 3.1 (10x Genomics) with STAR (version 2.7.2b) to process raw sequencing data in fastq format and align to mouse genome assembly GRCm38 (mm10). Seurat suite version 3.0 was used for downstream quality check, cell and gene feature filtering, and unsupervised clustering of cells. Seurat function 'FindClusters' with parameters "resolution = 0.8" and "dims.use = 1:20" were adopted in unsupervised clustering. R package "scMCA" to define cell types in mouse based on single-cell digital expression in Mouse Cell Atlas v2.0 (http://bis.zju.edu.cn/MCA/). Marker genes across all clusters or between two clusters were identified using Seurat 'FindMarkers' with parameter "min.pct=0.5". Bulk tissue data was analyzed using HISAT2 RNA-sequencing alignment software (version 2.1). Raw counts data were normalized using variance stabilizing transformation (VST) method implemented in R/Bioconductor "DEseq2" package. Normalization was performed using R/Bioconductor "DEseq2" package. Sample and gene clustering were performed using R/Bioconductor package 'ComplexHeatmap', and "Seurat" function "DoHeatmap". We used R package "rWikiPathways" for pathway analysis of cluster markers or DEGs between experiment conditions. Threshold-free pathway analysis based on the differential expression rank of all genes was performed using R package "rWikiPathways" or Gene Set Enrichment Analysis (GSEA) software (version 4.1). For statistical analyses of other experiment results, Prism 8.0.1 software was used.

For manuscripts utilizing custom algorithms or software that are central to the research but not yet described in published literature, software must be made available to editors and reviewers. We strongly encourage code deposition in a community repository (e.g. GitHub). See the Nature Portfolio guidelines for submitting code & software for further information.

Data

Policy information about availability of data

All manuscripts must include a data availability statement. This statement should provide the following information, where applicable:

- Accession codes, unique identifiers, or web links for publicly available datasets
- A description of any restrictions on data availability
- For clinical datasets or third party data, please ensure that the statement adheres to our policy

The raw reads for scRNA-seq in fastq format have been deposited in Sequence Read Archive (SRA) database under accession number SRP335961 [https://www.ncbi.nlm.nih.gov/sra/?term=SRP335961]. The processed data and meta data that support the findings of this study have been deposited in the Gene Expression Omnibus (GEO) under accession number GSE183545 [https://www.ncbi.nlm.nih.gov/geo/query/acc.cgi?acc=GSE183545]. The raw reads data for bulk tissue RNA-seq were deposited in Sequence Read Archive (SRA) database under BioProject accession number PRJNA749378 [https://www.ncbi.nlm.nih.gov/bioproject/?term=PRJNA749378], and the raw expression data and normalized data were deposited in Gene Expression Omnibus (GEO) under accession number GSE183657 [https://www.ncbi.nlm.nih.gov/geo/query/acc.cgi?acc=GSE183657]. Source data are provided as a Source Data file with this paper. All other data are available in the article and its supplementary files or from the corresponding author upon request.

Human research participants

Policy information about studies involving human research participants and Sex and Gender in Research.

Reporting on sex and gender	N/A
Population characteristics	N/A
Recruitment	N/A
Ethics oversight	N/A

Note that full information on the approval of the study protocol must also be provided in the manuscript.

Field-specific reporting

Please select the one below that is the best fit for your research. If you are not sure, read the appropriate sections before making your selection.

☒ Life sciences ☐ Behavioural & social sciences ☐ Ecological, evolutionary & environmental sciences

For a reference copy of the document with all sections, see nature.com/documents/nr-reporting-summary-flat.pdf

Life sciences study design

All studies must disclose on these points even when the disclosure is negative.

Sample size	No sample size calculation was performed using any statistical method. Sample size (or number of repeats) was chosen based on what is common in the field, and what was practical to do. Sample size was determined to be adequate based on the reproducibility between independent experiments.
Data exclusions	No data were excluded from the analyses.
Replication	All experiments were replicated independently and all experiments were reliably reproduced. We cross verified the validity of data independently by more than three mice models, and both in vivo and in vitro which were repeated three or more independent times. We

strongly believe our data will be reproducible.

Randomization

Age and sex-matched animals were used in all experiments. For evaluating the effect of antibody in PF in vivo, humanized Pear1 mice were randomly assigned to different groups according to the body weight.

Blinding

The investigators were blinded during data collection and/or analysis.

Reporting for specific materials, systems and methods

We require information from authors about some types of materials, experimental systems and methods used in many studies. Here, indicate whether each material, system or method listed is relevant to your study. If you are not sure if a list item applies to your research, read the appropriate section before selecting a response.

Materials & experimental systems

n/a	Involved in the study	
☐	☒	Antibodies
☐	☒	Eukaryotic cell lines
☒	☐	Palaeontology and archaeology
☐	☒	Animals and other organisms
☒	☐	Clinical data
☒	☐	Dual use research of concern

Methods

n/a	Involved in the study	
☒	☐	ChIP-seq
☐	☒	Flow cytometry
☒	☐	MRI-based neuroimaging

Antibodies

Antibodies used

Details for antibodies used in western blotting (WB), immunofluorescence (IF) , flow cytometry are provided provided below:
1. Phospho-Smad2 (Ser465/467)/Smad3 (Ser423/425) (D27F4) Rabbit mAb CST #8828 WB 1:1K
2. Phospho-p38 MAPK (Thr180/Tyr182) Antibody CST #9211 WB 1:1K
3. Phospho-SAPK/JNK (Thr183/Tyr185) (81E11) Rabbit mAb CST #4668 WB 1:1K
4. Phospho-p44/42 MAPK (Erk1/2) (Thr202/Tyr204) (D13.14.4E) XP® Rabbit mAb CST #4370 WB 1:2K
5. Phospho-Akt (Ser473) (D9E) XP® Rabbit mAb CST #4060 WB 1:2K
6. PTEN (138G6) Rabbit mAb CST #9559 WB 1:1K
7. Phospho-PTEN (Ser380/Thr382/383) (44A7) Rabbit mAb CST #9549 WB 1:1K
8. PP1 antibody (E-9) santa sc-7482 WB 1:500 IP "1-2 µg per 100-500 µg of total protein "
9. Mouse PEAR1 Antibody R&D AF7607 WB 1:1K
10. Human PEAR1 Antibody R&D AF4527 WB 1:1K
11. Peroxidase AffiniPure Goat Anti-Mouse IgG (H+L) Jackson 115-035-003 WB 1:5K
12. Peroxidase AffiniPure Rabbit Anti-Goat IgG (H+L) Jackson 305-035-003 WB 1:1W
13. Peroxidase AffiniPure Goat Anti-Rabbit IgG (H+L) Jackson 111-035-003 WB 1:1W
14. Peroxidase AffiniPure Donkey Anti-Sheep IgG (H+L) Jackson 713-035-003 WB 1:5K
15. anti-GAPDH antibody Yeasen 30201ES WB 1:1W
16. 488-Goat Anti-Armenian hamster IgG H&L abcam ab173003 IF 1:500
17. Anti-Podoplanin / gp36 antibody abcam ab11936 IF 1:500
18. Anti-Collagen IV antibody abcam ab6586 IF 1:400
19. Anti-Ki67 antibody abcam ab15580 IF 1:100
20. Goat anti-Rabbit IgG (H+L) Highly Cross-Adsorbed Secondary Antibody, Alexa Fluor 546 Invitrogen A11035 IF 1:500
21. Mouse PDGF R alpha Antibody R&D AF1062 IF 1:100
22. Donkey anti-Goat IgG (H+L) Cross-Adsorbed Secondary Antibody, Alexa FluorTM 594 Invitrogen A11058 IF 1:200
23. Donkey anti-Rabbit IgG (H+L) Highly Cross-Adsorbed Secondary Antibody, Alexa Fluor™ 488 Invitrogen A21206 IF 1:400
24. Anti-alpha smooth muscle Actin antibody abcam ab5694 IF 1:100
25. BV421 Rat Anti-Mouse CD326（G8.8） BD biosciences 563214 Flow cytometry 1:100
26. PE Rat Anti-Mouse CD45（30-F11） BD biosciences 553081 Flow cytometry 1:100
27. FITC Rat Anti-Mouse CD31（390） BD biosciences 558738 Flow cytometry 1:100
28. "PE/Cyanine7 anti-mouse CD31 Antibody（390 ） " biolegend 102418 Flow cytometry 1:100
29. CD140a (PDGFRA) Monoclonal Antibody (APA5), PE, eBioscience™ Invitrogen 12-1401-81 Flow cytometry 1:100
30. PerCP-Cy™5.5 Rat Anti-Mouse CD45（30-F11） BD biosciences 561869 Flow cytometry 1:100
31. Human PEAR1 Alexa Fluor® 647-conjugated Antibody（492621） R&D FAB4527R Flow cytometry 1:100
32. BD Pharmingen™ FITC Rat Anti-Mouse CD62P/P-selectin（RB40.34） BD biosciences 553744 Flow cytometry 1:100
33. Integrin alphaIIbbeta3 (GPIIb/IIIa, CD41/CD61)-PE（JON/A） BD biosciences M023-2 Flow cytometry 1:50
34. The monoclonal antibody for activating human or mouse PEAR1 was generated by immunizing BABL/c or Pear1-/- mice with human or mouse PEAR1 ECD, respectively. Then one antibody for human PEAR1 was humanized by CDR graft and named LF2. The antibody for mouse PEAR1 named LF1.

Validation

1. Phospho-Smad2 (Ser465/467)/Smad3 (Ser423/425) (D27F4) Rabbit mAb. Rabbit monoclonal antibody. Species Reactivity: Human, Mouse, Rat, Monkey. Application: WB. https://www.cellsignal.cn/products/primary-antibodies/phospho-smad2-ser465-467-smad3-ser423-425-d27f4-rabbit-mab/8828?site-search-type=Products&N=4294956287&Ntt=%238828&fromPage=plp&_requestid=7493216
2. Phospho-p38 MAPK (Thr180/Tyr182) Antibody. Rabbit polyclonal antibody. Species Reactivity: Human, Mouse, Rat, Monkey, Pig. Application: WB,IP,IF. https://www.cellsignal.cn/products/primary-antibodies/phospho-p38-mapk-thr180-tyr182-antibody/9211?site-search-type=Products&N=4294956287&Ntt=%239211&fromPage=plp&_requestid=7494503
3. Phospho-SAPK/JNK (Thr183/Tyr185) (81E11) Rabbit mAb. Rabbit monoclonal antibody. Species Reactivity: Human, Mouse, Rat. Application: WB, IHC-P, IP. https://www.cellsignal.cn/products/primary-antibodies/phospho-sapk-jnk-thr183-tyr185-81e11-rabbit-mab/4668?site-search-type=Products&N=4294956287&Ntt=%234668&fromPage=plp&_requestid=7494607
4. Phospho-p44/42 MAPK (Erk1/2) (Thr202/Tyr204) (D13.14.4E) XP® Rabbit mAb. Rabbit monoclonal antibody. Species Reactivity: Human, Mouse, Rat, Hamster, Monkey, Dog, Pig. Application: WB, IHC-P, IF, IP, Flow cytometry. https://www.cellsignal.cn/products/primary-antibodies/phospho-p44-42-mapk-erk1-2-thr202-tyr204-d13-14-4e-xp-rabbit-mab/4370?site-search-type=Products&N=4294956287&Ntt=%234370&fromPage=plp&_requestid=7494730
5. Phospho-Akt (Ser473) (D9E) XP® Rabbit mAb. Rabbit monoclonal antibody. Species Reactivity: Human, Mouse, Rat, Hamster, Monkey. Application: WB, IHC-P, IF, IP, Flow cytometry. https://www.cellsignal.cn/products/primary-antibodies/phospho-akt-ser473-d9e-xp-rabbit-mab/4060?site-search-type=Products&N=4294956287&Ntt=%234060&fromPage=plp&_requestid=7494864
6. PTEN (138G6) Rabbit mAb . Rabbit monoclonal antibody. Species Reactivity: Human, Mouse, Rat, Monkey. Application: WB, IHC-P, IP. https://www.cellsignal.cn/products/primary-antibodies/pten-138g6-rabbit-mab/9559?site-search-type=Products&N=4294956287&Ntt=%239559&fromPage=plp&_requestid=7494980
7. Phospho-PTEN (Ser380/Thr382/383) (44A7) Rabbit mAb. Rabbit monoclonal antibody. Species Reactivity: Human, Mouse, Rat, Monkey. Application: WB. https://www.cellsignal.cn/products/primary-antibodies/phospho-pten-ser380-thr382-383-44a7-rabbit-mab/9549?site-search-type=Products&N=4294956287&Ntt=%239549&fromPage=plp&_requestid=7495107
8. PP1 antibody (E-9). Mouse monoclonal antibody. Species Reactivity: Human, Mouse, Rat, Canine, Bovine, Porcine. Application: WB, IP, IF, IHC-P, ELISA. https://www.scbt.com/zh/p/pp1-antibody-e-9
9. Mouse PEAR1 Antibody. Sheep polyclonal antibody. Species Reactivity: Mouse. Application: WB, Flow cytometry. https://www.rndsystems.com/cn/products/mouse-pear1-antibody_af7607
10. Human PEAR1 Antibody. Goat polyclonal antibody. Species Reactivity: Human. Application: WB, Flow cytometry. https://www.rndsystems.com/cn/products/human-pear1-antibody_af4527
11. Peroxidase AffiniPure Goat Anti-Mouse IgG (H+L). https://www.jacksonimmuno.com/catalog/products/115-035-003
12. Peroxidase AffiniPure Rabbit Anti-Goat IgG (H+L). https://www.jacksonimmuno.com/catalog/products/305-035-003
13. Peroxidase AffiniPure Goat Anti-Rabbit IgG (H+L). https://www.jacksonimmuno.com/catalog/products/111-035-003
14. Peroxidase AffiniPure Donkey Anti-Sheep IgG (H+L). https://www.jacksonimmuno.com/catalog/products/713-035-003
15. anti-GAPDH antibody. Mouse monoclonal antibody. Species Reactivity: Human, Mouse, Rabbit, Frog, Fish, Chicken, Rat. Application: WB, ELISA, IHC, IF. https://www.yeasen.com/products/detail/881
16. 488-Goat Anti-Armenian hamster IgG H&L. https://www.abcam.cn/goat-armenian-hamster-igg-hl-alexa-fluor-488-ab173003.html
17. Anti-Podoplanin / gp36 antibody. Syrian hamster monoclonal antibody. Species Reactivity: Mouse. Application: IHC-P, IF. https://www.abcam.cn/podoplanin--gp36-antibody-rtd4e10-bsa-and-azide-free-ab11936.html
18. Anti-Collagen IV antibody. Rabbit polyclonal antibody. Species Reactivity: Mouse, Rat, Hamster, Cow, Dog, Human, Pig, Zebrafish, African green monkey, Chinese hamster, Syrian hamster. Application: ELISA, IHC-Fr, WB, IHC-P, IP, ICC/IF, IHC-FrFl, IHC-FoFr. https://www.abcam.cn/collagen-iv-antibody-ab6586.html
19. Anti-Ki67 antibody. Rabbit polyclonal antibody. Species Reactivity: Mouse, Human. Application: IHC-P, ICC, IF. https://www.abcam.cn/ki67-antibody-ab15580.html
20. Goat anti-Rabbit IgG (H+L) Highly Cross-Adsorbed Secondary Antibody, Alexa Fluor 546. https://www.thermofisher.cn/cn/zh/antibody/product/Goat-anti-Rabbit-IgG-H-L-Highly-Cross-Adsorbed-Secondary-Antibody-Polyclonal/A-11035
21. Mouse PDGF R alpha Antibody. Goat polyclonal antibody. Species Reactivity: Mouse. Application: WB, IHC, IF. https://www.rndsystems.com/cn/products/mouse-pdgf-ralpha-antibody_af1062
22. Donkey anti-Goat IgG (H+L) Cross-Adsorbed Secondary Antibody, Alexa FluorTM 594. https://www.thermofisher.cn/cn/zh/antibody/product/Donkey-anti-Goat-IgG-H-L-Cross-Adsorbed-Secondary-Antibody-Polyclonal/A-11058
23. Donkey anti-Rabbit IgG (H+L) Highly Cross-Adsorbed Secondary Antibody, Alexa Fluor™ 488. https://www.thermofisher.cn/cn/zh/antibody/product/Donkey-anti-Rabbit-IgG-H-L-Highly-Cross-Adsorbed-Secondary-Antibody-Polyclonal/A-21206
24. Anti-alpha smooth muscle Actin antibody. Rabbit polyclonal antibody. Species Reactivity: Mouse, Human. Application: WB, IHC-P, IF. https://www.abcam.cn/alpha-smooth-muscle-Actin-antibody-ab5694.html
25. BV421 Rat Anti-Mouse CD326（G8.8）. Rat monoclonal antibody. Species Reactivity: Mouse. Application: Flow cytometry. https://www.bdbiosciences.com/zh-cn/search-results?searchKey=563214
26. PE Rat Anti-Mouse CD45（30-F11）. Rat monoclonal antibody. Species Reactivity: Mouse. Application: Flow cytometry. https://www.bdbiosciences.com/zh-cn/search-results?searchKey=553081
27. FITC Rat Anti-Mouse CD31（390）. Rat monoclonal antibody. Species Reactivity: Mouse. Application: Flow cytometry. https://www.bdbiosciences.com/zh-cn/search-results?searchKey=558738
28. PE/Cyanine7 anti-mouse CD31 Antibody（390 . Rat monoclonal antibody. Species Reactivity: Mouse. Application: Flow cytometry. https://www.biolegend.com/en-us/products/pe-cyanine7-anti-mouse-cd31-antibody-3942
29. CD140a (PDGFRA) Monoclonal Antibody (APA5), PE, eBioscience™. Rat monoclonal antibody. Species Reactivity: Mouse. Application: Flow cytometry. https://www.thermofisher.cn/cn/zh/antibody/product/CD140a-PDGFRA-Antibody-clone-APA5-Monoclonal/12-1401-81
30. PerCP-Cy™5.5 Rat Anti-Mouse CD45（30-F11）. Rat monoclonal antibody. Species Reactivity: Mouse. Application: Flow cytometry. https://www.bdbiosciences.com/zh-cn/search-results?searchKey=561869
31. Human PEAR1 Alexa Fluor® 647-conjugated Antibody（492621）. Mouse monoclonal antibody. Species Reactivity: Human. Application: Flow cytometry. https://www.rndsystems.com/cn/products/human-pear1-alexa-fluor-647-conjugated-antibody-492621_fab4527r
32. BD Pharmingen™ FITC Rat Anti-Mouse CD62P/P-selectin（RB40.34）. Rat monoclonal antibody. Species Reactivity: Mouse. Application: Flow cytometry. https://www.bdbiosciences.com/zh-cn/search-results?searchKey=553744

33. Integrin alphaIIbbeta3 (GPIIb/IIIa, CD41/CD61)-PE（JON/A）. Rattus monoclonal antibody. Species Reactivity: Mouse. Application: Flow cytometry. https://www.labome.com/product/Emfret-Analytics/M023-2.html
34. The specificity of the LF1 and LF2 was validated by Elisa, flow cytometry detection of fibroblasts and platelets from KO and humanPear1-transgenic mice.

Eukaryotic cell lines

Policy information about cell lines and Sex and Gender in Research

Cell line source(s)	HFL1 and PF fibroblasts were purchased from ATCC (ATCC; CCL-153; ATCC; PCS-201-020). The HEK293S cell line was obtained directly from Professor Aiwu Zhou (Shanghai Jiao Tong University School of Medicine).Studies in HFL1 and PF fibroblasts were approved by the Institutional Review Board on Human Subjects Research and Ethics Committees (Shanghai Jiao Tong University School of Medicine).
Authentication	All the cell lines we used have been authenticated in our laboratory according to the morphology description at ATCC website.
Mycoplasma contamination	All cell lines were tested negative for mycoplasma contamination.
Commonly misidentified lines (See ICLAC register)	No commonly misidentified cell line was used in the study.

Animals and other research organisms

Policy information about studies involving animals; ARRIVE guidelines recommended for reporting animal research, and Sex and Gender in Research

Laboratory animals	1.C57BL/6 mice,Pear1tm1a/tm1a,male,8~12-week-old<br>2.C57BL/6 mice, Pear1f/f,male,8~12-week-old<br>3.C57BL/6 mice, Col1a2-creERPear1f/f,male,8~12-week-old<br>4.C57BL/6 mice, PF4-cre+Pear1f/f,male,8~12-week-old<br>5.C57BL/6 mice,human Pear1,male,8~12-week-old<br>6.C57BL/6 mice,wild type,male,8~12-week-old<br>7.BALB/c mice,wild type,male,8~12-week-old
Wild animals	No wild animals were used in this study.
Reporting on sex	Since IPF is a disease with a male predominance, the mice used in all experiments are male.
Field-collected samples	No field-collected samples were used in this study.
Ethics oversight	All animal experiments including euthanasia were in accordance with protocols approved by the Institutional Animal care and Use Committee (IACUC) of Shanghai Jiao Tong University School of Medicine. All the mice were housed under a SPF condition (12 hours-light/dark cycle, 50% relative humidity, and 22±2°C) with free access to normal laboratory diet (SZS9126, Xietong Pharmaceutical Bioengineering, China) and water and monitored by inspection twice each day.

Note that full information on the approval of the study protocol must also be provided in the manuscript.

Flow Cytometry

Plots

Confirm that:

- [x] The axis labels state the marker and fluorochrome used (e.g. CD4-FITC).
- [x] The axis scales are clearly visible. Include numbers along axes only for bottom left plot of group (a 'group' is an analysis of identical markers).
- [x] All plots are contour plots with outliers or pseudocolor plots.
- [x] A numerical value for number of cells or percentage (with statistics) is provided.

Methodology

Sample preparation	The fresh lungs of mice were cut and digested to prepare single lung cells, which were collected after centrifugation and filtered by 40 µm cell strainer. Cells were counted and stained with surface antibodies in FACS buffer, followed by incubation for 30 min on ice.
Instrument	CytoFLEX (Beckman Coulter).

Software	The data was collected by the CytoFLEX software and analyzed by FlowJo (v10.0.7).
Cell population abundance	A minimum of 10000 cells were collected for the population assay.
Gating strategy	The cell populations were identified by size and granularity in the FSC/SSC scatter. Dead cells and non-singlet cells were excluded by 7-amino-actinomycin D staining and the FSC-A versus FSC-H characteristics. The positive signal were defined compared to the staining of negative control samples or single channel staining.

☒ Tick this box to confirm that a figure exemplifying the gating strategy is provided in the Supplementary Information.
